# Survival and prognostic factors of early‐stage non‐small cell lung cancer in Central and Eastern Europe: A prospective cohort study

**DOI:** 10.1002/cam4.5791

**Published:** 2023-03-23

**Authors:** Mahdi Sheikh, Shama Virani, Hilary A. Robbins, Lenka Foretova, Ivana Holcatova, Vladimir Janout, Jolanta Lissowska, Marie Navratilova, Anush Mukeriya, Miodrag Ognjanovic, Beata Swiatkowska, David Zaridze, Paul Brennan

**Affiliations:** ^1^ Genomic Epidemiology Branch International Agency for Research on Cancer (IARC/WHO) Lyon France; ^2^ Department of Cancer Epidemiology & Genetics Masaryk Memorial Cancer Institute Brno Czech Republic; ^3^ Department of Public Health and Preventive Medicine, Second Faculty of Medicine Charles University Prague Czech Republic; ^4^ Department of Oncology 2nd Medical Faculty & University Hospital Motol Prague Czech Republic; ^5^ Faculty of Medicine Palacky University Olomouc Czech Republic; ^6^ Department of Cancer Epidemiology and Prevention M. Sklodowska‐Curie National Research Institute of Oncology Warsaw Poland; ^7^ Department of Clinical Epidemiology N.N. Blokhin National Medical Research Centre of Oncology Moscow Russia; ^8^ International Organization for Cancer Prevention and Research Belgrade Serbia; ^9^ Department of Environmental Epidemiology Nofer Institute of Occupational Medicine Poland

**Keywords:** alcohol, death, mortality, neoplasm, smoking, tobacco

## Abstract

**Background:**

Although early diagnosis and surgical resection of the tumor have been shown to be the most important predictors of lung cancer survival, long‐term survival for surgically‐resected early‐stage lung cancer remains poor.

**Aims:**

In this prospective study we aimed to investigate the survival and prognostic factors of surgically‐resected early‐stage non‐small cell lung cancer (NSCLC) in Central and Eastern Europe.

**Methods:**

We recruited 2052 patients with stage I‐IIIA NSCLC from 9 centers in Russia, Poland, Serbia, Czech Republic, and Romania, between 2007–2016 and followed them annually through 2020.

**Results:**

During follow‐up, there were 1121 deaths (including 730 cancer‐specific deaths). Median survival time was 4.9 years, and the 5‐year overall survival was 49.5%. In the multivariable model, mortality was increased among older individuals (HR for each 10‐year increase: 1.31 [95% CI: 1.21–1.42]), males (HR:1.24 [1.04–1.49]), participants with significant weight loss (HR:1.25 [1.03–1.52]), current smokers (HR:1.30 [1.04–1.62]), alcohol drinkers (HR:1.22 [1.03–1.44]), and those with higher stage tumors (HR stage IIIA vs. I: 5.54 [4.10 – 7.48]). However, education, chronic obstructive pulmonary diseases (COPD), and tumor histology were not associated with risk of death. All baseline indicators of smoking and alcohol drinking showed a dose‐dependent association with the risk of cancer‐specific mortality. This included pack‐years of cigarettes smoked (*p*‐trend = 0.04), quantity of smoking (*p*‐trend = 0.008), years of smoking (*p*‐trend = 0.010), gram‐days of alcohol drank (*p*‐trend = 0.002), frequency of drinking (*p*‐trend = 0.006), and years of drinking (*p*‐trend = 0.016).

**Conclusion:**

This study shows that the 5‐year survival rate of surgically‐resected stage I‐IIIA NSCLC is still around 50% in Central and Eastern Europe. In addition to non‐modifiable prognostic factors, lifetime patterns of smoking and alcohol drinking affected the risk of death and disease progression in a dose‐dependent manner in this population.

## 1 INTRODUCTION

In 2020, an estimated 2.2 million people globally were diagnosed with lung cancer, and 1.79 million died of this disease, making it the leading cause of cancer death worldwide.^1^ Non‐small cell lung cancer (NSCLC) accounts for 85% of all lung cancer cases with cigarette smoking being the major risk factor.^2^ Following the successful implementation of tobacco control policies over the past decades, the incidence of lung cancer has been declining.^3^ However, lung cancer survival has only slightly improved and 5‐year survival remains below 25%.^4^, ^5^, ^6^, ^7^

Although early diagnosis and surgical resection of the tumor have been shown to be the most important predictors of lung cancer survival,^2^, ^8^, ^9^, ^10^ 5‐year survival for surgically resected early‐stage (I–IIIA) lung cancer remains poor.^11^, ^12^, ^13^, ^14^ There is limited information on prognostic factors among early‐stage surgically resected lung cancers.^15^, ^16^ Most available evidence regarding lung cancer survival originates from retrospective studies that are prone to selection and reporting bias,^14^, ^16^ or from registry data linkage studies that are prone to confounding and do not allow an accurate evaluation of the prognostic factors.^17^ Strong epidemiological data could help identify modifiable exposures with prognostic value and guide efforts to decrease the global burden of lung cancer.

We performed a large prospective study across 9 referral centers in 5 countries in eastern and central Europe to investigate the survival features and prognostic factors of early‐stage surgically resected NSCLC.

## 2 METHODS

### 2.1 Study population and design

This prospective study included patients from 9 medical centers across 5 countries in eastern and central Europe, with newly diagnosed surgically resected early‐stage (I, II, or IIIA) NSCLC, coded as C34 (site code) and as primary invasive (morphology code 3) according to the 3rd edition of the International Classification for Diseases in Oncology (ICD‐O‐3).^18^ From April 2007 to July 2016, participants were enrolled in Russia (departments of thoracic surgery in N.N. Blokhin National Medical Research Center of Oncology, and the City Clinical Oncological Hospital No.1 in Moscow). From October 2011 to June 2014, additional participants were enrolled in Czech Republic (Motol University Hospital in Prague, Masaryk Memorial Cancer Institute in Brno and University Hospital Olomouc in Olomouc), Romania (Marius Nasta Institute of Pneumology in Bucharest), Serbia (Clinical Centre of Serbia in Belgrade), and Poland (Institute of Tuberculosis and Lung Diseases in Warsaw, and Military Medical Academy in Lodz).

Participants were recruited after receiving a histological diagnosis of NSCLC but before receiving any local or systemic treatment. Upon enrolment, informed written consent was obtained from all participants. This study was approved by the local ethical committees of each study center and also the ethical committee of the International Agency for Research on Cancer.

### 2.2 Questionnaires and enrollment data

Upon enrolment all participants underwent an interview using a structured questionnaire to gather information on demographics, family and medical histories, and exposures and lifestyle habits. Participants were asked if they had been diagnosed with chronic health conditions including diabetes mellitus and hypertension, as well as any chronic obstructive pulmonary disorders (COPD) including chronic bronchitis and emphysema. Participants were also asked about their current weight and height, and their weight 2 years before diagnosis. We observed significant correlations between pre‐diagnostic weight loss and body mass index (BMI) at diagnosis (Table S1). Based on recent evidence,^19^, ^20^ we used the 2‐year pre‐diagnostic weight change for this analysis, rather than using BMI at enrolment. For each participant, we categorized weight change as “no weight loss”, “<5% weight loss”, or “≥5% weight loss”.

The questionnaire included questions about lifetime history of cigarette smoking, the starting and stopping ages to smoke cigarettes, as well as the frequency and amount of smoking cigarettes. Cigarette smokers were defined as participants who reported to have smoked at least 100 cigarettes through their lifetime and were categorized as “current smokers” or “former smokers.” Former smokers were defined as cigarette smokers who reported to have quit smoking at least 1 year before diagnosis. We calculated the cumulative cigarettes smoked in pack‐years (a pack includes 20 cigarettes) by multiplying the number of cigarette packs smoked per day by the number of years the participant had smoked.

The participants were asked about their lifetime history of drinking different types of alcoholic beverages. For each type of alcoholic beverage, the participants were further asked about the starting and stopping ages, as well as the frequency and amount of drinking the specified alcoholic beverage. Regular alcohol drinkers were defined as participants who reported to have drunk alcoholic beverages at least once per week for 1 year, who were further categorized as “current drinkers” and “former drinkers.” Former drinkers were defined as regular alcohol drinkers who reported to have quit drinking at least 1 year before diagnosis. We calculated the average daily consumption of alcoholic beverages using the reported frequency and amount of each alcoholic beverage. Then, we converted the average daily consumption of alcoholic beverages into milliliters of ethanol based on the ethanol content of alcoholic beverages in eastern and central European countries (Table S2) and multiplied this number by 0.816 to calculate the average daily consumption of ethanol by grams (for ethanol 1 milliliter = 0.816 grams). Finally, we calculated the cumulative amount of consumed pure alcohol in gram‐days by multiplying the grams of average daily consumption of ethanol by the number of years the participant had drank alcohol regularly.

The relevant medical, imaging, and pathology documents were reviewed by an expert local team to complete a separate questionnaire that included necessary information on the histopathological features of the tumor. The completed questionnaires and data underwent a quality control and quality assurance process by a central team. We categorized the participants based on their tumor histology as having “squamous cell carcinoma,” “adenocarcinoma,” and “neuroendocrine tumors.” The classification of tumor stage was performed upon diagnosis and before receiving any treatment for the current tumor, based on the 7th edition of the TNM classification system that is proposed by the American Joint Commission on Cancer (AJCC).^21^ For participants who were diagnosed before 2010, we converted tumor stage from the AJCC 6th edition TNM staging system to the AJCC 7th edition (Tables S3 and S4).

### 2.3 Follow‐up process

Patients were followed twice per year. Active and passive follow‐up processes were used to determine vital status, tumor progression (recurrence and metastasis), treatments, and causes of death. Active follow‐up was performed by contacting the patients, their families, their responsible physicians when applicable, and medical record review. Passive follow‐up was performed by linkage to the national cancer and death registries in each country. The primary and secondary causes of death were recorded based on the 10th Revision of the International Statistical Classification of Diseases and Related Health Problems.^22^

### 2.4 Statistical analysis

We used the Kaplan–Meier method and log‐rank tests to calculate and compare unadjusted survival probabilities. We used Cox proportional hazards regression models to estimate the probabilities of overall survival and progression‐free survival and identify their determinants. To assess the probability of lung‐cancer specific survival and its determinants, we used Fine‐Gray competing‐risks regression models, accounting for death from other causes as the competing event.

In all models the entry time was defined as the date at which the participant was diagnosed with NSCLC, and the exit time was defined as the end date of follow‐up time. For assessing the probability of overall survival and hazards of all‐cause mortality, date of death from any cause was set as the end of follow‐up time, while date of the last contact was set as the censoring date for patients who were alive at the last contact (through June 7, 2020). For assessing the probability of progression‐free survival, date of death from any cause or date of tumor progression, whichever occurred first was set as the end of follow‐up time, while date of the last contact was set as the censoring date for patients who were alive and did not have any documented tumor progression at the last contact. For assessing the probability of lung cancer‐specific mortality and its hazards, date of death from lung cancer was set as the end of follow‐up time for the event of interest, date of death from any other cause was set as the end of follow‐up time for the competing event, and date of the last contact was set as the censoring date for patients who were alive at the last contact.

The multivariable models were adjusted for study location, age at diagnosis, sex, education, amount of weight loss in the 2 years before diagnosis, history of COPD, history of chronic general health conditions, smoking status at diagnosis, regular‐alcohol drinking status at diagnosis, tumor histology, and tumor stage at diagnosis. All models were also stratified by year of diagnosis, chemotherapy, and radiotherapy after diagnosis. The proportional hazards assumption was tested using Schoenfeld's global test, which was met for all variables in the multivariable models, except for tumor stage which showed time‐varying effects and was therefore treated as a time‐varying covariate.^23^

We also evaluated the effects of different baseline indicators of cigarette smoking/drinking alcohol on NSCLC survival by replacing the smoking/drinking variables in the main models with variables that indicate cumulative cigarettes smoked/alcohol drank, intensity of smoking/drinking, and duration of smoking/drinking.

We also assessed the dose–response associations of different smoking and alcohol drinking indicators with risk of the study outcomes by assigning consecutive integers to the categories of each exposure indicator and calculating the *p* value for trend by treating these variables as continues variables in the adjusted models.

All statistical analyses were two‐sided and performed using Stata statistical software version 14 (Stata Corporation).

## 3 RESULTS

### 3.1 Descriptive statistics

Of the initial 2291 patients recruited into this study, 239 were excluded because of having incomplete data for variables used for downstream analysis, leaving 2052 patients eligible for analysis. Most patients were recruited in Russia (55%), a quarter were recruited in Poland (25%), and the remainder were recruited in Serbia, Czech Republic, and Romania (20%). The mean age at diagnosis was 63.5 years (SD 8.7 years), and most participants were male (71%). At the time of diagnosis, 41% had a diagnosis of hypertension and/or type 2 diabetes and 21% had COPD. Significant weight loss (5% or greater) in the 2 years prior to diagnosis was reported in 19% of participants; 54% of participants were current smokers, 32% were current alcohol drinkers, and presented with squamous cell carcinoma (46%) or adenocarcinoma (44%). Approximately half of the patients presented at stage I (51%) (Table 1).

**TABLE 1:** Estimates of crude survival rates of European patients with surgically resected early‐stage non‐small cell lung cancer by demographical and clinical characteristics.

Characteristics	*N* (%)	Median survival time (years)	*p*‐value^a^	3‐year survival (%)	5‐year survival (%)
Total					
	2052 (100)	4.9		62.9	49.5
Country^b^			0.67		
Russia	1122 (55)	5.1		62.5	49.8
Poland	508 (25)	4.6		62.5	47.3
Serbia	265 (13)	4.4		65.2	47.0
Czech Republic	84 (4)	—		66.9	—
Romania	73 (3)	—		59.7	—
Sex			**<0.001**		
Male	1461 (71)	4.1		58.9	44.9
Female	591 (29)	6.8		72.7	60.0
Education			0.053		
Primary school	686 (33)	4.2		59.2	45.7
High school	831 (41)	5.2		64.3	51.7
University	535 (26)	5.0		65.2	50.4
Weight loss (in the past 2 years)			**<0.001**		
No weight loss	431 (21)	5.8		67.0	53.8
Lost <5% of weight	1221 (60)	4.9		63.2	49.5
Lost ≥5% of weight	400 (19)	3.6		56.9	43.8
Smoking status at diagnosis			**<0.001**		
Never smoker	360 (18)	6.8		74.5	61.3
Former smoker	587 (28)	4.5		58.8	47.6
Current smoker	1105 (54)	4.3		61.2	46.3
Regular alcohol drinking at diagnosis			**<0.001**		
Never drinker	1070 (52)	5.7		67.2	53.9
Former drinker	324 (16)	4.0		56.1	43.6
Current drinker	658 (32)	4.1		59.0	44.8
Tumor histology			**0.006**		
Squamous cell carcinoma	952 (46)	4.4		60.3	46.6
Adenocarcinoma	891 (44)	5.4		66.7	52.9
Neuroendocrine tumors	209 (10)	4.5		58.0	46.2
Tumor stage			**<0.001**		
I‐A	504 (25)	8.4		78.9	63.0
I‐B	530 (26)	6.3		69.8	57.2
II‐A	334 (16)	4.9		61.9	49.2
II‐B	234 (11)	3.6		55.9	40.3
III‐A	450 (22)	2.0		40.7	28.7

*Note:* Bold values indicate statistical significance at the *p* < 0.05 level.

^a^ Log‐rank test *p* value.

^b^ Maximum follow‐up time for participants from Czech Republic and was 4.1 years and at the latest follow‐up more than 50% of patients were alive. Therefore median survival time and 5‐year survival rates could not be estimated for participants that were recruited from these countries.

### 3.2 Survival characteristics

The median survival time for all patients was 4.9 years, with variation across demographic and clinical characteristics. Better overall survival was observed in females (*p* < 0.001), those who did not smoke (*p* < 0.001), did not drink alcohol (*p* < 0.001), did not lose weight in the last 2 years (*p* < 0.001), were diagnosed with adenocarcinoma (*p* = 0.006), and diagnosed at the earliest stages (*p* < 0.001) (Table 1). Three‐ and 5‐year overall survival rates were 63% and 49%, while 3‐ and 5‐year progression‐free survival rates were 54% and 43%, respectively.

### 3.3 Clinical and demographic predictors of survival

**FIGURE 1:**
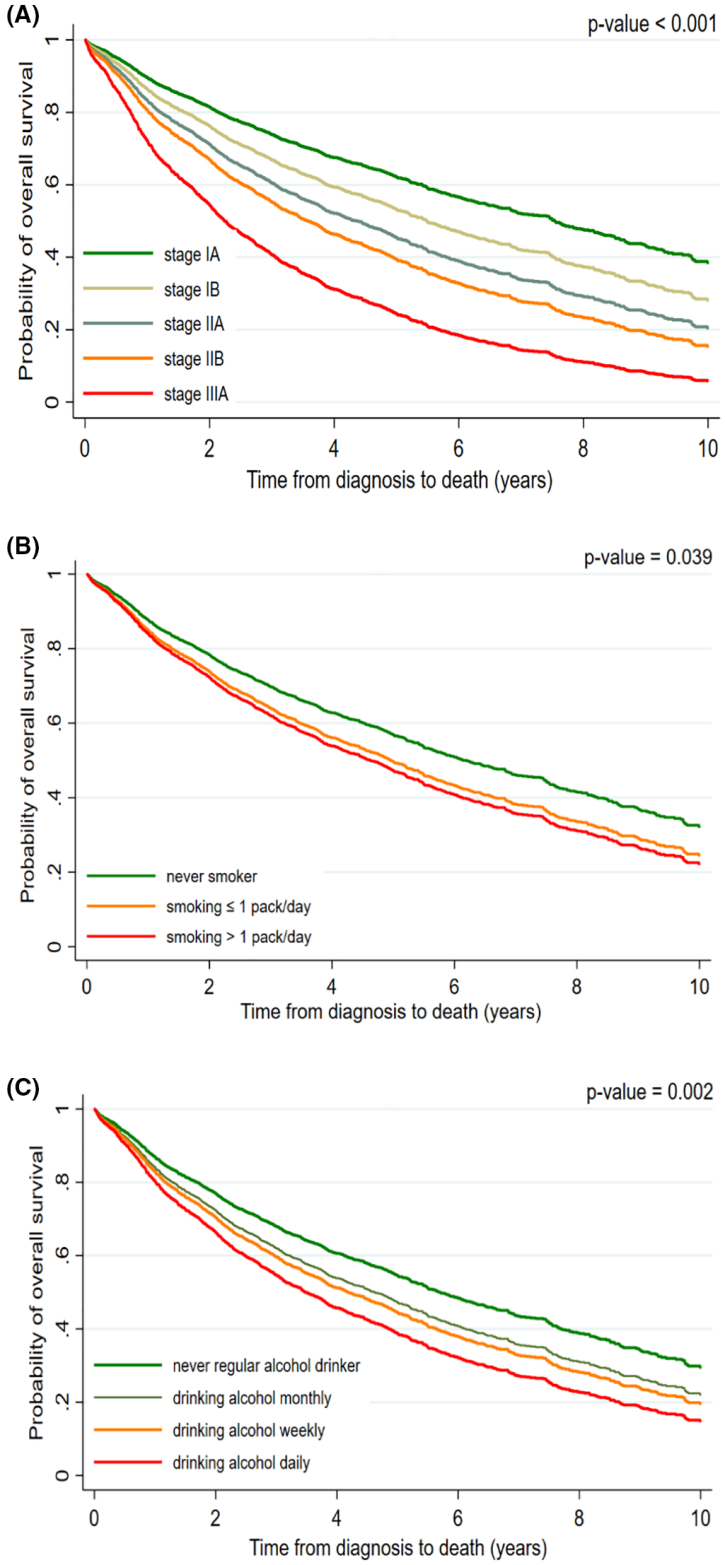
Adjusted overall survival estimates among European patients with surgically resected early stage (IA–IIIA) non‐small cell lung cancer by (A) stage at diagnosis, (B) smoking intensity at diagnosis, and (C) frequency of alcohol drinking at diagnosis. The figures are derived from proportional hazards regression models that included age, sex, study location, marital status, education, history of chronic respiratory disorders, history of chronic diseases, amount of weight loss, tumor histology, tumor stage, smoking, alcohol drinking, and received treatments (chemotherapy, radiotherapy).

In the multivariable regression model that was adjusted for potential confounders and risk factors, the hazard of all‐cause mortality was increased among older individuals (HR for each 10‐year increase in age: 1.31 [95% CI: 1.21–1.42]), males (HR: 1.24 [1.04–1.49]), those who exhibited weight loss ≥5% (HR: 1.25 [1.03–1.52]), current smokers (HR: 1.30 [1.04–1.62]), and regular alcohol drinkers (former drinkers HR: 1.26 [1.05–1.54]; current drinkers HR: 1.22 [1.03–1.44]) (Table 2). Tumor stage was strongly associated with increased hazards of death in a dose‐dependent manner as stage increased, reaching a more than fivefold increase in the hazards of death among individuals diagnosed at stage IIIA (HR: 5.54 [4.10–7.48]) (Table 2, Figure 1).

**TABLE 2:** Association between different demographical and clinical factors with risk of different outcomes in European patients with surgically resected early stage non‐small cell lung cancer.

Characteristic	*N* (%)	Overall mortality	Cancer‐specific mortality	Disease progression^c^
		Adjusted HR (95% CI)^a^	Adjusted HR (95% CI)^a^ ^,^ ^b^	Adjusted HR (95% CI)^a^
Study location				
Russia	1,122 (55)	1	1	1
Poland	508 (25)	1.12 (0.88–1.43)	**0.46 (0.31–0.68)**	1.04 (0.83–1.31)
Serbia	265 (13)	0.96 (0.72–1.27)	0.72 (0.48–1.07)	1.05 (0.81–1.36)
Czech Republic	84 (4)	0.92 (0.55–1.54)	0.67 (0.37–1.24)	0.98 (0.62–1.55)
Romania	73 (3)	0.92 (0.60–1.42)	0.76 (0.36–1.56)	0.98 (0.67–1.45)
Age at diagnosis (years)				
Each 10‐year increase in age	—	**1.31 (1.21–1.42)**	**1.11 (1.01–1.22)**	**1.25 (1.16–1.35)**
Sex				
Female	591 (29)	1	1	1
Male	1461 (71)	**1.24 (1.04–1.49)**	1.10 (0.88–1.37)	1.15 (0.97–1.36)
Education level				
Primary school	686 (33)	1	1	1
High school	831 (41)	0.93 (0.80–1.08)	0.90 (0.75–1.09)	0.94 (0.81–1.08)
University	535 (26)	0.89 (0.76–1.05)	0.96 (0.79–1.17)	0.91 (0.78–1.07)
Chronic obstructive pulmonary disorders^d^				
		
No	1629 (79)	1	1	1
Yes	423 (21)	0.99 (0.84–1.15)	0.89 (0.74–1.07)	0.97 (0.84–1.13)
Chronic diseases^e^				
No	1213 (59)	1	1	1
Yes	839 (41)	0.88 (0.77–1.02)	0.96 (0.81–1.13)	**0.87 (0.77–0.99)**
Weight loss (in the past 2 years)				
No weight loss	431 (21)	1	1	1
Lost <5% of weight	1221 (60)	1.01 (0.86–1.19)	1.05 (0.86–1.27)	0.97 (0.83–1.13)
Lost ≥5% of weight	400 (19)	**1.25 (1.03–1.52)**	1.16 (0.91–1.46)	1.14 (0.95–1.37)
Smoking				
Never	360 (18)	1	1	1
Former	587 (28)	1.13 (0.89–1.43)	1.14 (0.86–1.52)	1.04 (0.84–1.30)
Current	1105 (54)	**1.30 (1.04–1.62)**	**1.33 (1.03–1.72)**	1.16 (0.94–1.43)
Regular alcohol drinking				
Never	1070 (52)	1	1	1
Former	324 (16)	**1.26 (1.05–1.53)**	1.19 (0.94–1.52)	**1.26 (1.05–1.52)**
Current	658 (32)	**1.22 (1.03–1.44)**	**1.25 (1.03–1.52)**	**1.18 (1.01–1.38)**
Tumor histology				
Squamous cell carcinoma	952 (46)	1	1	1
Adenocarcinoma	891 (44)	1.05 (0.92–1.21)	1.05 (0.88–1.25)	1.11 (0.97–1.27)
Neuroendocrine tumors	209 (10)	1.18 (0.95–1.46)	1.16 (0.87–1.53)	**1.22 (0.99–1.49)**
Tumor stage^f^				
IA	504 (25)	1	1	1
IB	530 (26)	**1.40 (1.05–1.88)**	**1.72 (1.21–2.52)**	**1.30 (1.00–1.69)**
IIA	334 (16)	**2.05 (1.47–2.86)**	**2.32 (1.56–3.46)**	**1.78 (1.32–2.40)**
IIB	234 (11)	**3.10 (2.18–4.41)**	**3.31 (2.16–5.07)**	**2.18 (1.59–2.98)**
IIIA	450 (22)	**5.54 (4.10–7.48)**	**5.31 (3.68–7.65)**	**3.85 (2.94–5.03)**

*Note:* Bold values indicate statistical significance at the *p* < 0.05 level.

Abbreviations: CI, confidence interval; HR, hazards ratio.

^a^ The adjusted models simultaneously include all variables as shown in the table and are stratified by year of enrolment and treatments received post‐surgery (chemotherapy, radiotherapy).

^b^ For this analysis death from any cause other than lung cancer was set as a competing event.

^c^ disease progression is defined as local recurrence, metastasis, or death.

^d^ Chronic obstructive pulmonary diseases include chronic bronchitis and emphysema.

^e^ Chronic diseases include hypertension and type 2 diabetes mellitus.

^f^ The hazards of the outcomes for higher‐stage tumors did not remain consistent over time and decreased annually by 18% (IIB) and 26% (IIIA) for overall mortality, by 13% (IIA) and 18% (IIB) and 22% (IIIA) for cancer‐specific mortality; and by 9% (IIB) and 21% (IIIA) for disease progression.

The multivariable competing risk regression model showed that the hazard of cancer‐specific mortality was higher among older individuals (HR for each 10‐year increase in age: 1.11 [1.01–1.22]), current smokers (HR: 1.11 [1.03–1.72]), current drinkers (HR: 1.25 [1.03–1.52]), and those with higher stage tumors (HR for stage IIIA: 5.31 [3.68–7.65]) (Table 2).

From the multivariable disease progression model, the hazard of disease progression was increased among older individuals (HR for each 10‐year increase in age: 1.25 [1.16–1.35]), regular alcohol drinkers (former drinkers HR: 1.26 [1.05–1.52]; current drinkers HR: 1.18 [1.01–1.38]), and neuroendocrine tumors (HR: 1.22 [0.99–1.49]). In contrast, having a chronic illness was associated with a lower hazard of disease progression (HR: 0.87 [0.77–0.99]). There was increasing hazards of progression with increasing tumor stage, reaching almost a fourfold increase in the hazards of disease progression among individuals diagnosed at stage IIIA (HR: 3.85 [2.94–5.03]) (Table 2).

### 3.4 Smoking and alcohol drinking characteristics associated with outcomes

Specific measures of smoking and drinking behaviors were assessed in lieu of smoking or drinking status, respectively, in the adjusted models. Upon adjustment for drinking status and other variables, cumulative amount of cigarettes smoked (HR_>40 pack/years_ = 1.26 [1.00–1.59]), quantity of smoking (HR_>1 pack/day_ = 1.31 [1.03–1.68]), and smoking duration (HR_>50 years_ = 1.41 [1.07–1.85]) were associated with increased hazards of overall death (Table 3, Figure 1). These associations were in a dose‐dependent manner for quantity of cigarettes smoked (*p*_trend_ = 0.039) and smoking duration (*p*_trend_ = 0.044). Similarly, the hazard of cancer‐specific mortality showed a dose‐dependent increase with increasing amounts of cumulative cigarettes smoked (*p*_trend_ = 0.040), quantity of smoking (*p*_trend_ = 0.008), and smoking duration (*p*_trend_ = 0.010) (Table 3). An increased hazard of disease progression was observed for patients who smoked for more than 50 years compared to non‐users (HR = 1.28 [1.00–1.66]), although the hazards increased with longer smoking durations (*p*_trend_ = 0.050) (Table 3).

**TABLE 3:** Association between smoking and alcohol drinking characteristics and risk of different outcomes in European patients with surgically resected early stage non‐small cell lung cancer.

Characteristic	*N* (%)	Overall mortality	Cancer‐specific mortality	Disease progression^c^
		Adjusted^a^ HR (95% CI)	Adjusted^a^ ^,^ ^b^ HR (95% CI)	Adjusted^a^ HR (95% CI)
Cumulative amount of cigarettes smoked				
None	360 (18)	1	1	1
≤40 pack/years	759 (37)	1.22 (0.97–1.52)	1.17 (0.91–1.51)	1.09 (0.88–1.34)
>40 pack/years	924 (45)	**1.26 (1.00–1.59)**	**1.31 (1.01–1.71)**	1.15 (0.93–1.43)
*p*_trend_		0.099	**0.040**	0.18
Quantity of cigarettes smoked				
None	360 (18)	1	1	1
≤1 pack/day	1080 (53)	1.22 (0.98–1.51)	1.18 (0.92–1.51)	1.11 (0.91–1.36)
>1 pack/day	603 (29)	**1.31 (1.03–1.68)**	**1.41 (1.07–1.87)**	1.13 (0.90–1.42)
*p*_trend_		**0.039**	**0.008**	0.35
Smoking duration				
None	360 (18)	1	1	1
≤40 years	825 (40)	1.21 (0.96–1.52)	1.16 (0.89–1.49)	1.06 (0.86–1.31)
>40–50 years	634 (31)	1.19 (0.94–1.51)	1.23 (0.94–1.62)	1.13 (0.90–1.40)
>50 years	226 (11)	**1.41 (1.07–1.85)**	**1.58 (1.14–2.19)**	**1.28 (1.00–1.66)**
*p*_trend_		**0.044**	**0.010**	**0.050**
Cumulative amount of alcohol drank				
None	1070 (52)	1	1	1
≤20 grams/day	574 (28)	**1.17 (1.00–1.38)**	1.16 (0.96–1.42)	**1.16 (0.99–1.36)**
>20 grams/day	397 (20)	**1.37 (1.13–1.65)**	**1.43 (1.14–1.78)**	**1.31 (1.09–1.57)**
*p*_trend_		**0.001**	**0.002**	**0.003**
Alcohol frequency				
None	1070 (52)	1	**1**	1
Less than once a month	554 (27)	**1.18 (0.99–1.40)**	**1.21 (0.99–1.48)**	1.15 (0.97–1.35)
Several days a week	343 (17)	**1.28 (1.05–1.56)**	1.22 (0.97–1.52)	**1.25 (1.04–1.52)**
Daily	85 (4)	**1.48 (1.06–2.06)**	**1.76 (1.19–2.59)**	**1.50 (1.11–2.03)**
*p*_trend_		**0.002**	**0.006**	**0.002**
Alcohol drinking duration				
None	1070 (52)	1	1	1
≤40 years	561 (28)	**1.22 (1.03–1.45)**	**1.22 (1.00–1.49)**	**1.21 (1.03–1.43)**
>40 years	410 (20)	**1.26 (1.04–1.52)**	**1.29 (1.03–1.62)**	**1.20 (1.00–1.44)**
*p*_trend_		**0.009**	**0.016**	**0.026**

*Note*: Data were missing on smoking duration for 7 ever smokers; on quantity of smoked cigarettes for 9 ever smokers; and on duration of alcohol drinking for 11 ever alcohol drinkers. Bold values indicate statistical significance at the *p* < 0.05 level.

Abbreviations: CI, confidence interval; HR, hazards ratio.

^a^ The models included age, sex, study location, marital status, education, history of chronic respiratory disorders, history of chronic diseases, amount of weight loss, tumor histology, tumor stage, the smoking status (for analyses including alcohol variables indicated), and alcohol drinking status (for analyses including smoking variables indicated). All models are stratified by year of enrolment and treatments received post‐surgery (chemotherapy, radiotherapy).

^b^ For this analysis death from any cause other than lung cancer was set as a competing event.

^c^ Disease progression is defined as local recurrence, metastasis, or death.

On adjustment for smoking status and other variables, cumulative amount of alcohol drank (*p*_trend_ ≤ 0.003 for all outcomes), drinking frequency (*p*_trend_ ≤ 0.006 for all outcomes), and years of drinking (*p*_trend_ ≤ 0.026 for all outcomes) were associated with increased hazard of overall mortality, cancer‐specific mortality, and disease progression, respectively, in a dose‐dependent manner (Table 3, Figure 1).

## 4 DISCUSSION

This international multicentric prospective study showed that the 5‐year survival rate for stage I–IIIA NSCLC is still poor in Central and Eastern Europe at 49.5%. Almost 60% of patients experience disease progression within 5 years of their diagnosis. In this population, baseline demographics and clinical features including older age, male gender, pre‐diagnostic weight loss, smoking, and alcohol intake were associated with a higher risk of mortality and disease progression after adjustment for lung cancer stage. Importantly, lifetime patterns of smoking and alcohol intake affected the survival of these patients in a dose‐dependent manner.

Our study shows that the 5‐year survival of patients with early stage (I–IIIA) NSCLC remains below 50% in Central and Eastern Europe. Poor survival is a shared feature of lung cancer across Europe, as shown in an analysis of the EUROCARE project that included data from 87 European cancer registries in 28 countries.^5^ In the EUROCARE project, the average 5‐year survival for lung cancer (stages I–IV) across Europe was estimated around 13%, between 1999 and 2007.^5^ Although our analysis only included surgically resected early‐stage NSCLC cases, stage at diagnosis consistently remained the most important predictor of NSCLC survival in all patient subgroups. The 5‐year survival rate ranged from 63% among patients with stage IA tumors to 28.7% in patients with stage IIIA tumors. This is consistent with findings from large‐scale retrospective studies in different parts of Europe (e.g., 5‐year survival rate for stage I lung cancer = 66.1%–72.4% in Switzerland and 56.6 in the United Kingdom; and for stage III: 16.5%–23.1% in Switzerland and 12.6% in the United Kingdom).^4^, ^24^, ^25^ Given the fact that a large proportion of lung cancer cases are still diagnosed at advanced stages,^24^, ^25^, ^26^ international efforts are needed to facilitate earlier diagnosis, including by screening of high‐risk populations.^10^, ^26^, ^27^, ^28^

In our study, patients with NSCLC who were older, male, and current smokers had lower survival. The duration and intensity of smoking increased the hazards of all‐cause and cancer‐specific death, in a dose‐dependent manner. The prognostic value of age and sex for lung cancer survival has been previously demonstrated.^29^, ^30^, ^31^ However, current literature shows conflicting results for smoking status at the time of diagnosis. While many studies have shown prognostic effects for smoking status at diagnosis,^32^, ^33^, ^34^, ^35^ some studies did not find any effect for smoking status at the time of diagnosis on the survival of these patients.^36^, ^37^, ^38^ These conflicting findings might be partly due to the changes in postdiagnosis smoking behavior among current smokers that has not been accounted for in different studies.^39^ We recently showed that quitting smoking after the diagnosis of lung cancer could reduce the risk of death by 30% among current smokers.^39^ A recent review by Gemine and Lewis has identified different mechanisms that could underlie the negative effects of smoking continuation on lung cancer survival that include the effect of tobacco smoke and its carcinogenic compounds on the promotion of tumor growth, cellular damage, genetic mutations, immunosuppression, increasing resistance to and complications from the available treatments, and enhancing tumor recurrence and other comorbidities that can potentially increase the risk of mortality in lung cancer patients.^16^

There is also conflicting evidence on the relationship between alcohol consumption and lung cancer survival, ranging from studies that showed beneficial effects for consuming low amounts of alcohol^40^ to studies that showed negative effects of alcohol consumption for only short‐term outcomes including postsurgical complications,^41^, ^42^ but not‐long‐term outcomes,^43^ and those that showed the negative effects on long‐term outcomes.^44^ In the current study, all indicators of alcohol consumption, including the duration of lifetime consumption, frequency of consumption, and cumulative amount of consumed alcohol, were associated with a dose‐dependent increase in risk of all‐cause and cancer‐specific mortality, and disease progression compared to never consumption of alcohol. There is growing evidence of complex interactions between alcohol use/abuse, and the antitumor immune response, tumor growth, and metastasis.^45^ However, more prospective and mechanistic research is needed to investigate the complex relationship between alcohol and cancer survival.^45^

Many studies have previously shown the inverse relationship between BMI at diagnosis and lung cancer survival.^46^, ^47^ Our results that show the significant inverse correlation between pre‐diagnostic weight loss with BMI and tumor stage at diagnosis. We also show the prognostic effects of pre‐diagnostic weight loss on NSCLC survival and disease progression, which supports the growing evidence that pre and post diagnosis weight changes might be more reliable prognostic factors for lung cancer survival than weight or BMI at the time of diagnosis.^19^, ^48^ The observed effect of BMI at diagnosis might partly be a consequence of the preexisting illness resulting in weight loss and distorting the true relationship between BMI and the risk of death and therefore clinicians should be alerted against misinterpreting the current evidence that obesity might be “good” or “protective” for cancer patients.^19^, ^20^, ^49^

The strengths of our study are its multicentric prospective design, large sample size, high data quality, and long‐term follow‐up which allowed assessment of multiple outcomes among early‐stage surgically resected lung cancer patients across several countries. One limitation is that the exposures were based on self‐reported data. However, due to the prospective nature of this study, any measurement errors in exposures are likely to be non‐differential by the studied outcomes. Due to the observational nature of the study, we did not intervene in patients' management process and ascertained the cause of death and disease progression using the available death certificates and patients' medical records. Consequently, some misclassifications in the cancer‐specific mortality and disease progression are inevitable due to the variation in physicians' therapeutic and diagnostic approaches and patient's adherence to the follow‐up visits. However, death status is not likely to be affected by these measurements error.

In conclusion, this study shows that the survival of early‐stage surgically resected NSCLC is still poor in Central and Eastern Europe. In addition to non‐modifiable prognostic factors (e.g., age, gender, tumor stage), lifetime patterns of smoking and alcohol drinking affected the risk of death and disease progression in a dose‐dependent manner. Therefore, to decrease the burden of lung cancer, implementation of early detection and treatment strategies should be accompanied by supporting behavioral changes.

## AUTHOR CONTRIBUTIONS

**Mahdi Sheikh:** Conceptualization (lead); formal analysis (lead); investigation (lead); methodology (lead); project administration (equal); supervision (lead); visualization (lead); writing – original draft (lead); writing – review and editing (lead). **Shama Virani:** Formal analysis (equal); investigation (equal); methodology (equal); visualization (equal); writing – original draft (equal); writing – review and editing (equal). **Hilary A Robbins:** Methodology (equal); visualization (equal); writing – review and editing (equal). **Lenka Foretova:** Project administration (equal); resources (equal); writing – review and editing (equal). **Ivana Holcatova:** Project administration (equal); resources (equal); writing – review and editing (equal). **Vladimir Janout:** Project administration (equal); resources (equal); writing – review and editing (equal). **Jolanta Lissowska:** Project administration (equal); resources (equal); writing – review and editing (equal). **Marie Navratilova:** Project administration (equal); resources (equal); writing – review and editing (equal). **Anush Mukeriya:** Project administration (equal); resources (equal); writing – review and editing (equal). **Miodrag Ognjanovic:** Project administration (equal); resources (equal); writing – review and editing (equal). **Beata Swiatkowska:** Project administration (equal); resources (equal); writing – review and editing (equal). **David Zaridze:** Project administration (equal); resources (equal); writing – review and editing (equal). **Paul Brennan:** Conceptualization (equal); data curation (equal); investigation (equal); methodology (equal); supervision (equal); writing – review and editing (equal).

## FUNDING INFORMATION

This work was funded by the International Agency for Research on Cancer (IARC).

## CONFLICT OF INTEREST STATEMENT

No potential conflict of interest was reported by the authors.

## ETHICS STATEMENT

This study was performed in line with the principles of the Declaration of Helsinki. Upon enrolment, informed written consent was obtained from all participants. This study was approved by the local ethical committees of each study center and also the ethical committee of the International Agency for Research on Cancer (IARC).

## DISCLAIMER

Where authors are identified as personnel of the International Agency for Research on Cancer/World Health Organization, the authors alone are responsible for the views expressed in this article and they do not necessarily represent the decisions, policy, or views of the International Agency for Research on Cancer/World Health Organization.

## Supporting information


**Supporting information S1.** Supplementary material

## Data Availability

The datasets analyzed during the current study are available from the corresponding author upon reasonable request.
