# Supplementary material for: Survival and prognostic factors of early‐stage non‐small cell lung cancer in Central and Eastern Europe: A prospective cohort study
Source: Cancer Med. 2023 Mar 23;12(9):10563–74. doi: 10.1002/cam4.5791 (PMC10225235; doi:10.1002/cam4.5791)
Supplement: Supplementary file 1 — Supporting information S1. Supplementary material [file CAM4-12-10563-s001.docx]

| **Table S1.** Associations between body mass index (BMI) at diagnosis and weight loss during the two years before diagnosis. | | | | | |
| --- | --- | --- | --- | --- | --- |
| **Weight loss amount** | **BMI Groups ^1^ [N (%)]** | | | | **P value ^2^** |
|  | Underweight | Normal | Overweight | Obese | <0.001 |
| Not lost weight | 0 (0) | 124 (15) | 191 (24) | 116 (32) |  |
| Lost <5% of weight | 17 (40) | 512 (60) | 486 (61) | 206 (56) |  |
| Lost ≥5% of weight | 25 (60) | 212 (25) | 117 (15) | 46 (12) |  |
| **1** BMI was calculated by dividing the weight in kilograms by height in meters squared. Participants were categorized based on their BMI as underweight for BMI < 18.5, as normal for 18.5 ≤ BMI < 25, as overweight for 25 ≤ BMI < 30, and as obese for BMI ≥ 30.  **2** P value of the chi square test | | | | | |

| **Table S2:** size of glasses/bottles and concentrations of ethanol in the study locations. | | | | | | |
| --- | --- | --- | --- | --- | --- | --- |
|  | **Central Europe** | | | **Russia** | | |
| **Type of alcohol** | Unit | Vol per unit | Ethanol content (%) | Unit | Vol per unit | Ethanol content (%) |
| **Beer** | bottle | 500 ml | 5 | bottle | 500 ml | 5 |
| **Wine** | Glass | 100 ml | 12 | Glass | 150 ml | 12 |
| **Aperitif/desert wines** | Glass | 100 ml | 18 | Glass | 150 ml | 18 |
| **Homemade spirits** | Shot | 20 ml | 40 | Grams | 1 ml | 30 |
| **Commercial spirits** | Shot | 20 ml | 40 | Grams | 1 ml | 40 |

**References:**

1. IARC Working Group on the Evaluation of Carcinogenic Risks to Humans. 1988. Alcohol Drinking. (IARC Monographs on the Evaluation of Carcinogenic Risks to Humans, No. 44). Lyon, France: International Agency for Research on Cancer.
2. IARC Working Group on the Evaluation of Carcinogenic Risks to Humans. 2010. Alcohol Consumption and Ethyl Carbamate. (IARC Monographs on the Evaluation of Carcinogenic Risks to Humans, No. 96). Lyon, France: International Agency for Research on Cancer.

| **Table S3.** Differences between the 6th and 7th editions of American Joint Committee on Cancer **(**AJCC) methods of assigning TNM for lung cancer staging (differences are shown in green) | | | |
| --- | --- | --- | --- |
| **TNM 7th edition (AJCC cancer staging manual 2010)** | | **TNM 6th edition (AJCC cancer staging manual 2002)** | |
| **T** |  | **T** |  |
| TX | Primary tumor cannot be assessed, or tumor proven by the presence of malignant cells in sputum or bronchial washings but not visualized by imaging or bronchoscopy | Tx | Primary tumor cannot be assessed, or tumor proven by the presence of malignant cells in sputum or bronchial washings but not visualized by imaging or bronchoscopy |
| T0 | No evidence of primary tumor | T0 | No evidence of primary tumor |
| Tis | Carcinoma in situ | Tis | Carcinoma in situ |
| T1 | Tumor 3 cm or less in greatest dimension, surrounded by lung or visceral pleura, without bronchoscopic evidence of invasion more proximal than the lobar bronchus (i.e., not in the main bronchus) | T1 | Tumor 3 cm or less in greatest dimension, surrounded by lung or visceral pleura, without bronchoscopic evidence of invasion more proximal than the lobar bronchus (i.e., not in the main bronchus |
| T1a | Tumor 2 cm or less in greatest dimension |  |  |
| T1b | Tumor more than 2 cm but 3 cm or less in greatest dimension |  |  |
| T2 | Tumor more than 3 cm but 7 cm or less or tumor with any of the following features (T2 tumors with these features are classified T2a if 5 cm or less): involves main bronchus, 2 cm or more distal to the carina; invades visceral pleura (PL1 or PL2); associated with atelectasis or obstructive pneumonitis that extends to the hilar region but does not involve the entire lung | T2 | Tumor with any of the following features of size or extent :  More than 3cm in greatest dimension  Involves main bronchus, 2cm or more distal to the carina  Invades the visceral pleura  Associated with atelectasis or obstructive pneumonitis that extends to the hilar region bubt does not involve the entire lung |
| T2a | Tumor more than 3 cm but 5 cm or less in greatest dimension |  |  |
| T2b | Tumor more than 5 cm but 7 cm or less in greatest dimension |  |  |
| T3 | Tumor more than 7 cm or one that directly invades any of the following: parietal pleural (PL3), chest wall (including superior sulcus tumors), diaphragm, phrenic nerve, mediastinal pleura, parietal pericardium; or tumor in the main bronchus (less than 2 cm distal to the carina a but without involvement of the carina); or associated atelectasis or obstructive pneumonitis of the entire lung or separate tumor nodule(s) in the same lobe | T3 | Tumor of any size that directly invades any of the following: chest wall (including superior sulcus tumors), diaphragm, mediastinal pleura, parietal pericardium; or tumor in the main bronchus less than 2 cm distal to the carina, but without involvement of the carina; or associated atelectasis or obstructive pneumonitis of the entire lung |
| T4 | Tumor of any size that invades any of the following: mediastinum, heart, great vessels, trachea, recurrent laryngeal nerve, esophagus, vertebral body, carina, separate tumor nodule(s) in a different ipsilateral lobe | T4 | Tumor of any size that invades any of the following: mediastinum, heart, great vessels, trachea, esophagus, vertebral body, carina, ; or separate tumor nodule(s) in the same lobe ; or tumor with malignant pleural effusion |
|  |  |  |  |
| N |  | N |  |
| NX | Regional lymph nodes cannot be assessed | NX | Regional lymph nodes cannot be assessed |
| N0 | No regional node metastases | N0 | No regional node metastases |
| N1 | Metastases to ipsilateral peribronchial and/or ipsilateral hilar node(s) and intrapulmonary nodes, including involvement by direct extension | N1 | Metastasis to ipsilateral peribronchial and/or ipsilateral hilar lymph node(s) and intrapulmonary nodes, including involvement by direct extension of the primary tumor |
| N2 | Metastases to ipsilateral mediastinal and/or subcarinal lymph node(s) | N2 | Metastasis to ipsilateral mediastinal and/or subcarinal lymph node(s) |
| N3 | Metastases in contralateral mediastinal, contralateral hilar, ipsilateral or contralateral scalene, or supraclavicular node(s) | N3 | Metastasis in contralateral mediastinal, contralateral hilar, ipsilateral or contralateral scalene, or supraclavicular lymph node(s) |
|  |  |  |  |
| M |  | M |  |
|  |  | MX | Distant Metastasis cannot be assessed |
| M0 | No distant metastasis | M0 | No distant metastasis |
| M1 | Distant metastasis | M1 | Distant metastasis present (M1 includes seperate tumor nodule(s) in a different lobe (ipsilateral or controlateral) |
| M1a | Separate tumor nodule(s) in a contralateral lobe tumor with pleural nodules or malignant pleural (or pericardial) effusion |  |  |
| M1b | Distant metastasis (in extrathoracic organs) |  |  |

| **Table S4.** Differences between the 6th and 7th editions of the American Joint Committee on Cancer (AJCC) for staging lung cancer based on the TNM methods (the differences are shown in green) | | | | | | | |
| --- | --- | --- | --- | --- | --- | --- | --- |
| **TNM 7th edition (ajcc cancer staging manual 2010)** | | | | **TNM 6th edition (ajcc cancer staging manual 2002)** | | | |
| **Stage** | **T** | **N** | **M** | **Stage** | **T** | **N** | **M** |
| Occult carcinoma | Tx | N0 | M0 | Occult carcinoma | Tx | N0 | M0 |
| 0 | Tis | N0 | M0 | 0 | Tis | N0 | M0 |
| IA | T1a, T1b | N0 | M0 | IA | T1 | N0 | M0 |
| IB | T2a | N0 | M0 | IB | T2 | N0 | M0 |
| IIA | T2b | N0 | M0 | IIA | T1 | N1 | M0 |
|  | T1a, T1b | N1 | M0 |  |  |  |  |
|  | T2a | N1 | M0 |  |  |  |  |
| IIB | T2b | N1 | M0 | IIB | T2 | N1 | M0 |
|  | T3 | N0 | M0 |  | T3 | N0 | M0 |
| IIIA | T1a, T1b, T2a,  T2b | N2 | M0 | IIIA | T1 | N2 | M0 |
|  | T3 | N1, N2 | M0 |  | T2 | N2 | M0 |
|  | T4 | N0, N1 | M0 |  | T3 | N1, N2 | M0 |
| IIIB | Any T | N3 | M0 | IIIB | Any T | N3 | M0 |
|  | T4 | N2 | M0 |  | T4 | Any N | M0 |
| IV | Any T | Any N | M1 | IV | Any T | Any N | M1 |

For an overview on how the 7th edition of AJCC methods of assigning TNM and staging for non-small cell lung cancer maps to the current 8th edition of AJCC staging system please visit the following links: <https://radiologyassistant.nl/chest/lung-cancer/tnm-classification-8th-edition>
